# Effect of Glutamine on Antioxidant Capacity and Lipid Peroxidation in the Breast Muscle of Heat-stressed Broilers via Antioxidant Genes and HSP70 Pathway

**DOI:** 10.3390/ani10030404

**Published:** 2020-02-29

**Authors:** Hong Hu, Liang Chen, Sifa Dai, Jiaqi Li, Xi Bai

**Affiliations:** 1 College of Animal Science, Anhui Science and Technology University, Chuzhou 233100, China; haiyanghh@163.com (H.H.); liyideji@sina.com (J.L.); 2 State Key Laboratory of Animal Nutrition, Institute of Animal Sciences, Chinese Academy of Agriculture Science, Beijing 100193, China; chenliang01@caas.cn; 3 Department of Pharmaceutical and Life Sciences, Jiujiang University, Jiujiang 332005, China; daisifa-004@163.com

**Keywords:** glutamine, heat stress, antioxidant capacity, lipid peroxidation, HSP70, antioxidants, meat quality, broiler

## Abstract

**Simple Summary:**

Broilers are heat stress sensitive animals. Heat stress can reduce the meat quality and increase the oxidative stress of broilers. The aim of this study was to investigate the effect of dietary supplementation with Glutamine (Gln) on meat quality, antioxidant capacity, lipid peroxidation, antioxidant genes, and the HSP70 gene and protein expression in broilers exposed to cyclic heat stress. Results showed that Gln improved the meat quality in heat-stressed broilers. In conclusion, our study suggested that dietary Gln supplementation alleviated antioxidant capacity and lipid peroxidation in the breast muscle of heat-stressed broilers by antioxidant genes and the HSP70 pathway.

**Abstract:**

This study investigated whether Glutamine (Gln) could be used as an additive to improve antioxidant capacity in the breast muscle of heat-stressed broilers. Two hundred and forty 22-day-old Arbor Acres broilers in the G1, G2, G3, and G4 groups (*n* = 60 each) were housed in a cyclic hot environment and fed the basal diet with 0%, 0.5%, 1.0%, and 1.5% Gln, respectively. Compared with the G1 group, dietary 1.5% Gln increased (*p* < 0.05) pH and b* values, but decreased (*p* < 0.05) L* cooking loss, drip loss, and water loss rate in breast meat of heat-stressed broilers. Malondialdehyde levels in the breast muscle were lower (*p* < 0.05) in 1.0% and 1.5% Gln groups than that of the heat-stress group. Compared with the G1 group, dietary 1.5% Gln increased (*p* < 0.05) catalase (CAT), glutathione, glutathione peroxidase (GSH-Px,) and total antioxidant capacity in the breast muscle of heat-stressed broilers. Furthermore, the CAT, GSH-Px, HSP70 mRNA expression levels, and HSP70 protein expression levels were increased (*p* < 0.05) in the G3 and G4 groups compared with the G1 group. In sum, Gln alleviated antioxidant capacity and lipid peroxidation in the breast muscle of heat-stressed broilers through antioxidant genes and HSP70 pathways.

## 1. Introduction

Broilers are heat stress sensitive animals [1]. When the ambient air temperature increases to an extent that the broilers’ body cannot adjust, the body cannot expel heat in time, resulting in a heat stress reaction. Heat stress can increase body metabolism, produce large amounts of free radicals, cause lipid peroxidation, damage proteins and DNA, and lead to oxidative stress, which decreases the meat quality [2,3,4]. Due to intensive, industrial feeding and global warming, the degree of heat stress on poultry is increasing, especially in the hot and humid south in summer, which seriously restricts the development of the poultry industry [5,6,7]. Therefore, it is of great significance to find feasible methods to enhance the heat resistance of the body.

Heat shock protein 70 (HSP70) is one of the most important proteins in the heat shock protein (HSP) family [8,9]. The level of HSP70 is very low under non-stress condition, and the heat stress transcription factor (HSF) exists in the cytoplasm in the form of monomers. Under heat and other stress conditions, HSF combines with the heat shock promoter element (HSE) to activate the transcriptional synthesis of HSP70 and increase the expression of HSP70. The level of HSP70 is positively correlated with temperature under high-temperature stress [10,11,12,13]. HSP70 is considered to be an important protective molecule that measures the functional state and anti-stress ability of cells under stress [14].

Glutamine (Gln), a precursor of glutamate, is involved in many metabolic pathways [15]. When the body is stressed, the level of Gln decreases, which suppresses the function of the body. The addition of Gln enhances the body’s resistance to stress [16,17]. Our previous studies revealed that Gln can improve meat quality and effectively alleviate the adverse reactions caused by heat stress in broilers [18,19]. In addition, some studies have shown that Gln can increase the expression of HSP70 in the body and reduce cellular and oxidative damage [20,21,22]. It is speculated that Gln can induce and enhance the expression of HSP70, thus increasing the ability to resist heat stress.

Although it has been shown that Gln has a positive effect in alleviating heat stress, there is little research on the relationship between Gln and HSP70 expression in broilers under high temperature conditions [23]. Therefore, in this study, we determined whether Gln can enhance the anti-oxidative stress ability of the breast muscle by increasing the expression of antioxidant genes and HSP70 in broilers exposed to cyclic heat stress.

## 2. Materials and Methods 

### 2.1. Birds, Experimental Design and Sample Collection

The experiment was performed according to the guidelines for the care and use of experimental animals of the Ministry of Science and Technology of the People’s Republic of China (Approval Number 2006-398) and approved by the Animal Care and Use Committee of Anhui Science and Technology University. Two hundred and forty 22-day-old Arbor Acres broilers were obtained from the farm in the Anhui Science and Technology University. Six replicates (10 broilers per replicate) of four heat-stressed groups including G1, G2, G3, and G4 were employed. The birds in the G1, G2, G3, and G4 groups were housed in a hot environment and fed the basal diet with 0%, 0.5%, 1.0%, and 1.5% Gln, respectively. The hot environment was 34 °C (humidity: 60–70%) for 8 h (09:00–17:00) and 24 °C (humidity: 45–55%) during rest time (17:00-09:00). The basal diet (Table 1) was designed based on the NRC [24], Bai et al. [19] and Hu et al. [25]. The duration of the heat exposure was 21 days. At 42 days of age, 18 broilers/group (3 broilers/replicate) were selected and sacrificed to collect the breast muscle samples for further analysis.

### 2.2. Meat Quality Analysis

The pH of breast meat was detected using a pH device (Mettler-Toledo, Greisensee, Switzerland) 45 min after slaughter, as described by Hu et al. [25]. The meat color of the breast was detected using a Minolta colorimeter (Konica-Minolta, Tokyo, Japan) according to lightness (L*), redness (a*), and yellowness (b*). The cooking loss (CL), drip loss (DL) and water loss rate (WLR) were measured as described by Hu et al. [25]. The breast meat was weighed (W_Initial_), steamed in a pot for 30 min, and then reweighted (W_Final_). The equation of CL is: CL (%) = (W_Initial_ − W_Final_)/W_Initial_ × 100. To measure DL, the breast meat was weighed (W_Initial_) and placed in a plastic bag at 4 °C. After 24 h, the meat sample was reweighted (W_Final_). The equation of DL is: DL (%) = (W_Initial_ − W_Final_)/W_Initial_ × 100. To measure WLR, the breast was weighed (W_Initial_) and placed in 18 layers of filter paper. Then, the sample was pressed at 2000 psi for 60 s. The meat sample was reweighed immediately (W_Final_). The equation of WLR is: WLR (%) = (W_Initial_ − W_Final_)/W_Initial_ × 100.

### 2.3. Measurement of Redox State in the Breast Muscle

The 10% homogenate of breast muscle was prepared under cold conditions. The superoxide dismutase (SOD), catalase (CAT), glutathione (GSH), glutathione peroxidase (GSH-Px), total antioxidant capacity (T-AOC), and malondialdehyde (MDA) were detected by commercial kits (Jiancheng Institute of Bioengineering, Nanjing, China) with a spectrophotometer (Jingke company, Shanghai, China).

### 2.4. Measurement of HSP70 Levels in Breast Muscle

The HSP70 level in the breast muscle was detected using a chicken HSP70 enzyme-linked immunosorbent assay (ELISA) kit which was purchased from Jiancheng Institute of Bioengineering (Nanjing, China) with a Thermo Scientific Multiskan GO (Massachusetts, USA). 

### 2.5. Quantitative Real-Time PCR (qRT-PCR)

Total RNA was extracted from breast muscle according to the manufacturer’s instructions of the TianGen RNAprep pure tissue kit (Beijing, China). The isolated total RNA was measured by a spectrophotometer (Eppendorf, Hamburg, Germany) and reverse transcribed with the PrimeScript™ RT reagent Kit with gDNA Eraser (Takara, Dalian, China). 

Relative mRNA expression levels of *HSP70*, *GSH-Px*, *CAT*, *SOD*, and *β-actin* (housekeeping gene) were quantified by qRT-PCR with primers (Table 2) in accordance with Hu et al. [25]. The qRT-PCR was performed on the Roche LightCycler 480 II (Basel, Switzerland) using TB Green^®^ Premix Ex Taq™ (Takara, Dalian, China) according to the manufacturer’s protocol. The reaction system and program were similar to the method of Zhang et al. [26]. Relative mRNA expression of the target gene was quantified by the 2^−ΔΔCT^ method [27].

### 2.6. Statistical Analysis

The statistical analyses were performed using the SPSS statistical package (version 18.0) with a one-way analysis of variation (ANOVA). Duncan’s test was used to evaluate the difference among the groups. The data were expressed as mean ± SEM and *p* < 0.05 was regarded as significant. Linear and quadratic trends were used to detect the influences of different levels of Gln in the diet.

## 3. Results

### 3.1. Meat Quality

Table 3 shows the meat quality of breast in broilers exposed to heat stress. Compared with the G1 group, the b* increased (*p* < 0.05), but the DL (*p* < 0.05) decreased in the G2 group; the b* and pH were increased (*p* < 0.05), but the L* and DL were decreased (*p* < 0.05) in the G3 group; the b* and pH were increased (*p* < 0.05), but the L*, DL, WLR, and CL were decreased (*p* < 0.05) in the G4 group. Furthermore, the b*, L*, pH, DL, WLR and CL of the breast meat in the Gln-supplemented groups showed a liner dose response (*p* < 0.05).

### 3.2. MDA in the Breast Muscle

**Figure 1:**
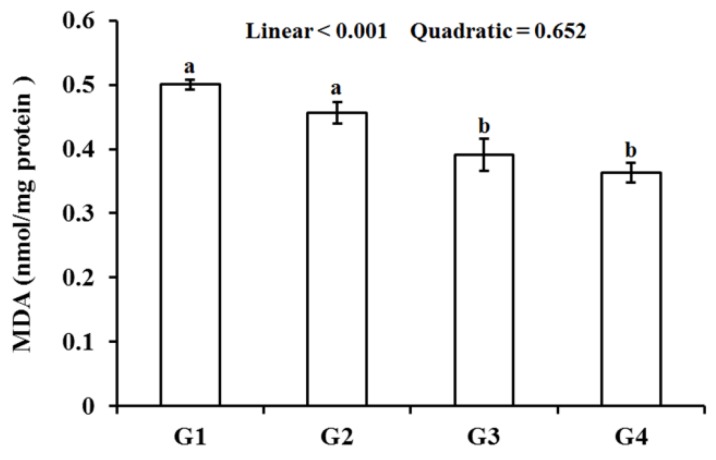
Effect of Gln on MDA of the breast muscle in heat-stressed broilers. a, b: Values in columns with different letters differ significantly (*p* < 0.05). Broilers in the G1, G2, G3, and G4 groups were housed in the hot environment and fed the basal diet with 0, 0.5%, 1.0%, and 1.5% Gln, respectively.

As shown in Figure 1, the MDA decreased (*p* < 0.05) in the G3 and G4 groups compared with the G1 group. The MDA of the breast muscle in the Gln-supplemented groups showed a liner dose response (*p* < 0.05).

### 3.3. SOD, CAT, GSH, GSH-Px, and T-AOC Levels in the Breast Muscle

Table 4 shows the redox status of breast in broilers exposed to heat stress. Compared with the G1 group, the T-AOC increased (*p* < 0.05) in the G2 group, the GSH and T-AOC increased (*p* < 0.05) in the G3 group, and the CAT, GSH, GSH-Px and T-AOC increased (*p* < 0.05) in the G4 group. The CAT, GSH GSH-Px and T-AOC of the breast muscle in the Gln-supplemented groups showed a liner dose response (*p* < 0.05). Furthermore, the CAT of the breast muscle in the Gln-supplemented groups showed a quadratic dose response (*p* < 0.05).

### 3.4. Gene Expression of Antioxidant Enzymes in the Breast Muscle

**Figure 2:**
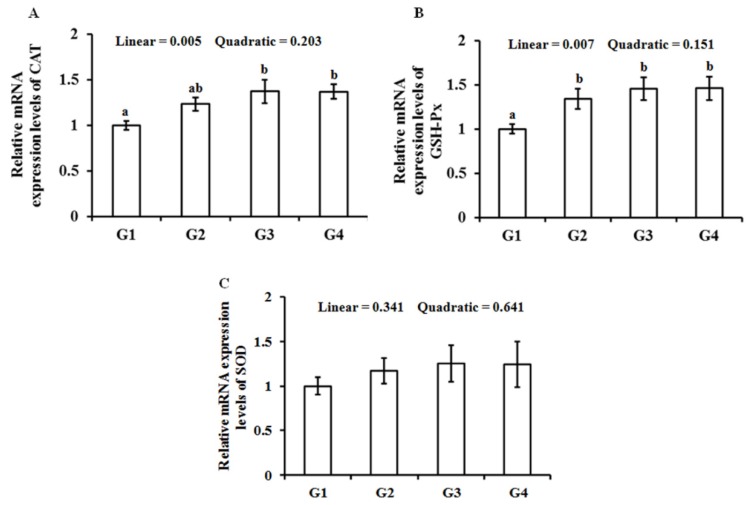
Effect of Gln on CAT (**A**), GSH-Px (**B**), and SOD (**C**) gene expression of the breast muscle in heat-stressed broilers. a, b: Values in columns with different letters differ significantly (*p* < 0.05). Broilers in the G1, G2, G3, and G4 groups were housed in the hot environment and fed the basal diet with 0%, 0.5%, 1.0%, and 1.5% Gln, respectively.

As shown in Figure 2, the *GSH-Px* mRNA expression level increased (*p* < 0.05) in the G2 group, and the *CAT* and *GSH-Px* mRNA expression levels increased (*p* < 0.05) in the G3 and G4 groups when compared with the G1 group. The *CAT* and *GSH-Px* mRNA expression levels showed a linear increase (*p* < 0.05) following the Gln supplementation.

### 3.5. Gene and Protein Expression Levels of HSP70 in the Breast Muscle

**Figure 3:**
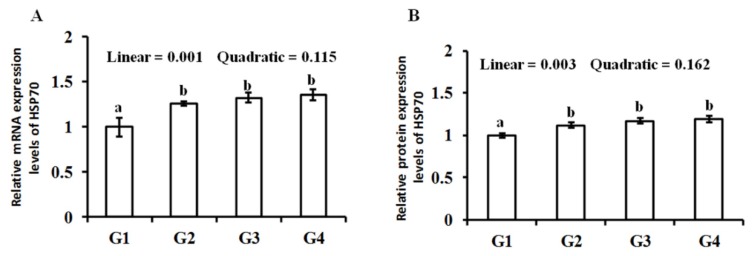
Effect of Gln *on HSP70* gene (**A**) and protein (**B**) expression of the breast muscle in heat-stressed broilers. a, b: Values in columns with different letters differ significantly (*p* < 0.05). Broilers in the G1, G2, G3, and G4 groups were housed in the hot environment and fed the basal diet with 0%, 0.5%, 1.0%, and 1.5% Gln, respectively.

As shown in Figure 3, the HSP70 mRNA and protein expression levers increased (*p* < 0.05) in the G2, G3, and G4 group when compared with the G1 group. The HSP70 mRNA and protein expression levers showed a linear increase (*p* < 0.05) following the Gln supplementation.

## 4. Discussion

With the improvement in people’s quality of life, there is an increasing demand for high-quality meat [4,25]. The main indexes of meat quality include muscle tenderness, water retention, color, and pH [28]. Several studies have showed that heat stress could reduce the meat quality of livestock and poultry [28,29]. Supplementation of exogenous Gln, during heat stress, can meet the requirements of tissue for Gln, improve the metabolism of the body, and effectively increase the pH of the muscle [18,30]. The present results also showed that dietary Gln significantly increased the pH value of breast muscle in stressed broilers. The meat pH not only directly reacted to muscle acidity, but also affected meat color, drip loss and other indicators [31]. In this experiment, the DL, WLR, CL and meat color of breast muscle were improved after the addition of Gln. Similar results were also found in Dai et al., which showed that dietary supplementation of 20 g/kg glutamine could significantly increase pH value and water content of chicken, and significantly decrease CL and L*of chicken [18]. These findings suggested that the addition of Gln could improve the negative effect of high temperature stress on meat quality.

Chicken meat is rich in unsaturated fatty acids, which is prone to lipid peroxide and produces pale soft exudative (PSE) meat [31]. MDA, a marker of lipid peroxidation, is a stable end product of lipid oxidation in broiler muscle [12,32,33]. Cheng et al. suggested that the decreased MDA reflect the reduction of oxidative stress damage caused by heat stress in the broiler muscle [34]. In the present study, dietary Gln could effectively reduce the level of MDA in the breast muscle, indicating that the lipid peroxidation was alleviated in heat-stressed broilers. 

It is easy to induce heat stress on broiler chicks in an environment of high temperature and high humidity [35,36]. Hot environments could stimulate the production of a large number of reactive oxygen species and free radicals in broiler breast muscle, which could become an important factor affecting meat quality [18,37,38]. SOD, GSH-Px, CAT, and GSH are the main antioxidants in the cells of the body, and their activity and content indirectly reflect the ability of the body to scavenge free radicals [12]. This study showed that the GSH-Px, CAT, GSH, T-AOC and T-AOC in the broiler breast muscle were significantly increased in the high temperature group after adding Gln. Furthermore, the supplementation of Gln raised the gene expression levels of GSH-Px and CAT in the breast muscle of heat-stressed broilers. Similarly, Hu et al. reported that dietary Gln improved antioxidant capacity and lipid peroxidation by activating the antioxidant gene expression in the thigh muscle of broiler exposed to cyclic heat stress [25].

HSP70 is distributed in various cell types, and its expression increases significantly under stress conditions [14,39]. It can enhance the tolerance of cells to stimulants, protect tissues, and reduce lipid peroxidation and DNA breakage induced by reactive oxygen species, thereby making the body adapt to heat and improve body tolerance [40]. Because of its physiological and pathological functions, HSP70 has become a hot spot in broiler science research. Several research articles indicated that there was an empirical relationship between oxidative stress and HSP70 expression [40,41]. Zhang et al. pointed out a raised *HSP70* transcriptional level when broilers were exposed to hot environments [12]. Parallel to the research of Gu et al., our results showed that the HSP70 mRNA and protein level in the breast muscle of heat-stressed broiler chickens increased significantly after the addition of Gln [42]. These results suggested that Gln could protect the cells by increasing the HSP70 level in the breast muscle and thus reducing the oxidative damage caused by heat stress.

## 5. Conclusions

The present study suggested that dietary Gln supplementation alleviated antioxidant capacity and lipid peroxidation in the breast muscle of heat-stressed broilers by antioxidant genes and the HSP70 pathway.

## Figures and Tables

**Table 1:** Composition of the basal diet ^1^.

Ingredients	% as Fed Basis	Chemical Composition	% as Fed Basis
Corn	58.5	ME (MJ/kg)	12.73
Soybean meal	32.0	Crude protein	20.30
Fish meal	2.0	Lysine	1.08
Starch	1.0	Methionine + cysteine	0.76
Soybean oil	3.5	Ca	0.89
CaHPO_4_·2H_2_O	1.5	Available P	0.42
Limestone	0.9		
Salt	0.3		
DL-Met	0.1		
Vitamin and trace mineral premix ^2^	0.2		
Total	100		

^1^ The basic diet was formulated according to the National Research Council (NRC) [24], Bai et al. [19] and Hu et al. [25]. ^2^ Provided per kilogram of diet: vitamin K: 2.0 mg; vitamin A: 10,000 IU; Fe (from Fe_2_(SO_4_)_3_): 80 mg; Zn (from ZnSO_4_), 40 mg; Cu (from CuSO_4_): 8 mg; Se (from Na_2_SeO_3_): 0.15 mg; vitamin B6: 3.0 mg; vitamin B12: 0.014 mg; vitamin E: 20 IU; cholecalciferol: 2600 IU; thiamin: 1.6 mg; riboflavin, 6.0 mg; folic acid: 0.8 mg; calcium pantothenate: 20 mg; biotin 0.12 mg; niacin: 30 mg; I (from IK): 0.35 mg; choline (as choline chloride), 500 mg.

**Table 2:** Specific sequences primers used in the qRT-PCR ^1^.

Gene	Primer (5′→3′)	Genbank Number
β-actin	F: TGCTGTGTTCCCATCTATCG R: TTGGTGACAATACCGTGTTCA	NM 205518
SOD	F: GGAGGAGTGGCAGAAGT R: TAAACGAGGTCCAGCAT	NM 205064
CAT	F: TATCCTTCCTGGTCTTTCTACAT R: CGCCATCTGTTCTACCTCC	NM 001031215.2
GSH-Px	F: AAGTGCGAGGTGAACGG R: CGGCGACCAGATGATGTAC	NM 001277853.1
HSP70	F: GGAGGACTTTGACAACCG R: CAAGCTGTACGCAGACG	NM_001006685.1

^1^ SOD: superoxide dismutase; GSH-Px: glutathione peroxidase; CAT: catalase; HSP70: Heat shock protein 70.

**Table 3:** Effect of Gln on meat quality of the breast meat in heat-stressed broilers.

Item	Treatment				SEM ^1^	*p*-Value	
	G1	G2	G3	G4		Linear	Quadratic
pH	5.905 ^a^	5.942 ^a^	6.063 ^b^	6.072 ^b^	0.019	<0.001	0.579
L*	46.007 ^a^	44.394 ^a^ ^b^	43.463 ^b^	43.246 ^b^	0.346	0.002	0.228
a*	4.104	4.394	4.494	4.548	0.087	0.074	0.496
b*	4.904 ^a^	5.677 ^b^	5.893 ^b^	5.913 ^b^	0.121	0.001	0.055
DL (%)	4.9 ^a^	4.7 ^b^	4.7 ^b^	4.5 ^b^	0.04	<0.001	0.561
WLR (%)	32.3 ^a^	31.0 ^a^	30.8 ^a^	28.8 ^b^	0. 4	0.001	0.621
CL (%)	35.9 ^a^	35.3 ^a b^	34.5 ^a b^	34.0 ^b^	0. 3	0.017	0.958

^a, b^: Values in rows with different letters differ significantly (*p* < 0.05). ^1^ SEM: standard error of the mean. Broilers in the G1, G2, G3, and G4 groups were housed in the hot environment and fed the basal diet with 0, 0.5%, 1.0%, and 1.5% Gln, respectively.

**Table 4:** Effect of Gln on antioxidant of the breast muscle in heat-stressed broilers.

Item	Treatment				SEM ^1^	*p*-Value	
	G1	G2	G3	G4		Linear	Quadratic
SOD (U/mg protein)	79.753	84.852	92.357	91.232	3.396	0.19	0.658
CAT (U/mg protein)	2.115 ^a^	2.340 ^a^ ^b^	2.874 ^a^ ^b^	3.105 ^b^	0.146	0.007	0.991
GSH (mg/g protein)	3.174 ^a^	4.083 ^a b^	5.392 ^b c^	6.324 ^c^	0.357	<0.001	0.983
GSH-Px (U/mg protein)	11.783 ^a^	16.187 ^a^ ^b^	15.963 ^a b^	18.456 ^b^	0.841	0.006	0.517
T-AOC (U/mg protein)	1.722 ^a^	3.084 ^b^	3.233 ^b^	2.930 ^b^	0.214	0.035	0.037

^a, b, c^: Values in rows with different letters differ significantly (*p* < 0.05). ^1^ SEM: standard error of the mean. Broilers in the G1, G2, G3, and G4 groups were housed in the hot environment and fed the basal diet with 0%, 0.5%, 1.0%, and 1.5% Gln, respectively.
